# Can carbon reduction policies promote sustainable construction development? Evidence from China’s green building market

**DOI:** 10.1371/journal.pone.0303149

**Published:** 2024-05-09

**Authors:** Wenjie Liu, Yuqing Chen, Peng Zhu, Jinjie Tong

**Affiliations:** 1 Business School, Jiangsu Open University, Nanjing, Jiangsu, China; 2 School of Management, Jiangsu University, Zhenjiang, Jiangsu, China; 3 Business School, Wuhan Qingchuan University, Wuhan, Hubei, China; 4 Jiangxi Institute of Science and Technology Information, Nanchang, Jiangxi, China; Krirk University, THAILAND

## Abstract

Carbon emissions have become a global challenge, and China, as the world’s largest developing country, has a serious emissions problem. Developing green buildings is an important way of reducing carbon emissions. China’s low-carbon city pilot policy may be an effective way of promoting green building development and reducing these emissions. This study uses the low carbon city pilot policy as a quasi-natural experiment and employs the staggered difference-in-differences method to investigate its impact on green building development. The results show that the low-carbon city pilot policy promotes green building development, and this policy promotes it by enhancing regional green innovation capacity, improving green total factor productivity at the firm and regional levels, and reducing the financing constraints of firms in the construction and real estate sectors. In addition, the promotion effect of the policy on green building development is stronger in western and non-resource-based regions and large-scale cities in China. This study contributes to the literature related to environmental policy, green building, and carbon emissions and supports the promotion of green building development and the reduction of carbon emissions.

## 1. Introduction

Climate change is a serious global challenge. Many mitigation strategies have been adopted to cope with the adverse effects of climate change. The construction industry has been criticized for contributing to high energy consumption and greenhouse gas emissions [1–3].According to the Fourth Assessment Report of the Intergovernmental Panel on Climate Change(IPCC) greenhouse gases from building activities account for approximately 27% of global carbon emissions [4]. It is estimated that by 2035, global carbon dioxide emissions from buildings will increase to 42.4 billion tons [5]. Given that it accounts for a disproportionately large share of total carbon emissions, reducing emissions in the building sector is an important way to achieve an overall reduction and realize green development [6–8]. Reducing energy consumption in the building sector can mitigate the negative impacts of building stock on the economic, social and natural environments [1,9] and is key to achieving the goals of the Paris Agreement [10]. Recognizing the challenge of reducing energy consumption and greenhouse gas emissions in the building sector, the development of green buildings is an important way to achieve green development in the building sector [11].

The World Green Building Council defines green buildings as buildings designed, constructed, or operated to have a positive impact on the climate and natural environment. Green buildings use technologies and materials that use fewer resources and lower carbon emissions during their design and construction and subsequent operation and maintenance [12,13]. They use environmentally friendly materials, combined with resource-saving and energy-reducing building concepts, to reduce energy consumption and carbon emissions associated with design, construction, operation, and maintenance, leading to healthier and more resource-efficient building models [14,15]. As such, they are an important way to realize a clean and green construction sector [16–18].The World Bank predicts that the contribution of building energy efficiency to the achievement of energy saving and emission reduction targets is about 70%, which means that green buildings will become an important trend in the future [19]. However, despite years of promotion and rapid technological advances, the development of green buildings is still limited and uncertain [20].

China, as the world’s largest construction market, emits more than 2.1 billion tons of carbon annually, of which the total emissions from the construction sector account for 46.5% [21]. The country’s huge building stock (over 60 billion square meters of floor space) consumes more than 1 billion tons of coal-equivalent primary energy, accounting for 22% of China’s total energy use and 20% of its energy-related CO2 emissions [22]. In the context of “carbon peaking and carbon neutrality,” conserving energy and reducing emissions in China’s construction sector has become a major issue [23]. Green building is an important driving force for China to realize clean and green development of the construction industry and to achieve the goal of “carbon peaking and carbon neutrality.” [17] It is predicted that carbon emissions from China’s urban and rural residential sectors will peak in 2046 and 2025, respectively [24]. Therefore, how to promote the green building development (GBD) is an urgent task that needs to be addressed by the government and policy makers.

The Chinese government has introduced numerous incentive and restraint policies to reduce emissions, among which its low-carbon city pilot policy (LCPP) is an important example. The LCPP, carried out in three batches in 2010, 2012, and 2017, aims to decarbonize city development by popularizing the concept of the low-carbon economy, adjusting industrial structures, and improving energy efficiency. As a result of the positive externalities of green buildings, the green building market faces market failure. As a result, it is difficult to realize the optimal supply of green buildings by relying on the market alone; government intervention–for example, in the form of the LCPP–could make up for this market failure, reduce carbon emissions, and promote green development. What is the impact of the LCPP on GBD and its mechanism? Addressing these questions is important for promoting GBD through green policies related to carbon emission reduction in China.

First, we offer a theoretical analysis of the LCPP’s impact on green building development and the effect mechanism. We then empirically examine the relationship between the two in the context of China by leveraging the LCPP as a quasi-natural experiment and employing a staggered difference-in-differences (DID) model. We carry out the parallel trend, placebo, and other policy shock tests and consider the heterogeneity of the treatment effect to assess robustness. We also construct indicators of regional green innovation, green total factor productivity at the regional and firm level, and firm financing constraints to empirically examine the mechanism by which LCPP affects GBD. In addition, we analyze city-level heterogeneity in terms of location, resource endowment, and scale of the impact of the LCPP on GBD.

The contribution of this study is threefold. First, this study enriches the literature related to green building. Research on green buildings mainly focuses on green building policies and regulations [17,25] and spatial distribution characteristics [26,27]. Some studies have focused on the impact of economic factors (e.g., incremental cost of construction, level of economy development, level of real estate development), technical factors (e.g., construction technology, energy conversion technology) and natural factors (e.g., soil) on GBD [15,28–30]. Public policy is also an important factor influencing GBD because of the market failure of the green building sector. This study, leveraging the quasi-natural experiment of China’s LCPP, is the first to examine the impact of LCPP on GBD, thereby enriching the research on the impact of government policies on GBD. Second, this paper enriches the study on the impact of public policies on GBD. Extant Studies have examined the impact of policies on green buildings from the perspectives of green finance, financial subsidies, and technical assistance [31–33]. This study matches prefecture- and firm-level data to examine the mechanism by which LCPP affects GBD, thereby offering theoretical evidence and policy references to better utilize the role of LCPP in promoting GBD. Third, this study contributes to the literature on the effect of government environmental policies. The existing literature on the effects of environmental policies has mostly focused on the impacts on corporate behavior and broader macroeconomic effects [34,35]; less attention has been given to the impact of the development of specific sectors. Further, this study analyzes the impact on GBD of governmental environmental regulatory policies, enriching studies related to the effects of environmental regulatory policies.

## 2. Literature review

Extant literature that studied the factors influencing green building are divided into three main categories. The first category is economic factors. On the one hand, regional economic factors closely affect the GBD. Economic growth can lead to the development of the construction industry, and bring market opportunities for green buildings [26]. Kok et al. found that regions with higher levels of economic development as well as well-established real estate markets have a higher degree of GBD [36]. This is because sustained economic growth creates an industrial agglomeration effect that incentivizes real estate developers to construct more green buildings [37]. In addition, market competition may accelerate GBD in the neighborhood and attract other developers to pursue green spillovers, as the premium return on green investments will signal to other real estate developers. On the other hand, the economic situation of firms is also an important factor affecting GBD. Jung et al. identified high additional costs as a major issue hindering the rapid development of green buildings [38]. Additional costs are unavoidable because green buildings must use innovative technologies and materials to achieve higher energy efficiency and environmental standards. This generates additional costs, which reduce the incentives for firms to develop green buildings. Shrestha and Pushpala argued that the additional and operational costs of green buildings are usually higher than those of conventional buildings, leading to a relatively low willingness of real estate developers to develop them [39]. Ge et al. assessed the perceptions of building developers towards sustainable buildings and found that the incremental costs of green buildings hindered their willingness to construct [40].

The second category is technical factors. The development and maintenance of green buildings involves complex technological issues such as technology adoption and energy performance transformation [41]. Green building technology is a decisive factor in realizing clean and green development in the construction industry [17,18]. Therefore, technological innovation is the key to achieving sustainable development in the green building industry [42]. Introducing green building technologies into the construction industry has become a key measure in the global sustainability strategy [17]. In addition, green building innovations can further improve the emission reduction effect of green buildings and thus promote cleaner production [26,43].

The third category is policy factors. The cost of developing green buildings is much higher compared to ordinary buildings. However, the benefits of green buildings are not fully capitalized in the final transaction price, resulting in a mismatch between costs and benefits. This will cause market failure and inhibit real estate companies from supplying green buildings [41]. In addition, the large upfront investment and long payback period of green buildings lead to low willingness of developers to construct green buildings. Therefore, the government should use policy incentives to promote GBD [31,38]. The positive role of public policy in promoting GBD has been widely demonstrated [44–46]. Chan et al. pointed out that policy incentives significantly contributed to the development of green buildings [32]. Kong and He comparatively analyzed the effects of supply-side policies and demand-side policies on green building technologies and found that demand-side policies did not significantly stimulate innovation, while supply-side green building policies effectively promoted GBD [25]. Karkanias et al. studied the green building market and found that insufficient policy incentives and the policy environment constrained GBD in Greek [47]. Matisoff et al. found that the government can influence GBD either as an investor or a user of buildings [31]. Zou et al. pointed out that financial subsidies can stimulate GBD in China [48]. Li et al. argued that the government should reasonably adjust the number of financial subsidies and improve the penalty mechanism, thus motivating real estate developers to actively apply for green buildings [49]. A study by Shen and Faure from a policy synergy perspective found that no single tool by itself is the best tool to promote green building, and therefore a smart mix needs to be designed to promote green building [50].

Among the policy factors, some studies have focused on the impact of carbon emission reduction policies on GBD. Although these studies have examined the impact of carbon emission reduction policies on corporate green innovation, corporate profits, corporate total factor productivity, regional economic growth, and foreign trade, there are fewer studies focused on the impact of carbon emission reduction policies on green buildings. Some studies focusing on this area have mainly examined the impact of green finance on GBD. Green finance aims to provide preferential loans and financing interest rate for cleaner production firms, thus motivating their green production and reducing carbon emissions [51,52]. Shen and Faure stated that environmental policies aim to internalize negative externalities associated with pollution and are also true for green buildings [50]. Debrah et al. argued that green buildings, although crucial for climate change mitigation, have a huge investment deficit [53]. Green credit can bridge the green building investment deficit as it can provide companies and projects with mortgages and project loans that facilitate the green transition. This can mitigate the lack of green building investment [54]. He et al. found that the synergistic effect of green bonds, green insurance, and financial subsidies can promote GBD [55].

## 3. Institutional background

### 3.1 Green building in China

Green building is defined as maximizing resource conservation, environmental protection, and pollution reduction and saving energy, land, and water through the entire life span of a building. It covers all stages of planning, design, construction, operation and management, demolition, and recycling during that life span and establishes a whole-process building-management system [12,13,15]. Green buildings can reduce or eliminate negative impacts on the climate and natural environment in their design, construction, or operation [56,57] and thus have received widespread attention around the world.

In China, the criteria for green buildings are set by the government: buildings that have received the green building evaluation label are generally considered to be green buildings. The Ministry of Housing and Urban-Rural Development of China (MOHURD) issued the first green building standard in June 2006, marking the beginning of GBD in China. In January 2013, the Green Building Action Plan was implemented, suggesting that China has increased GBD incentives [58].

**Fig 1:**
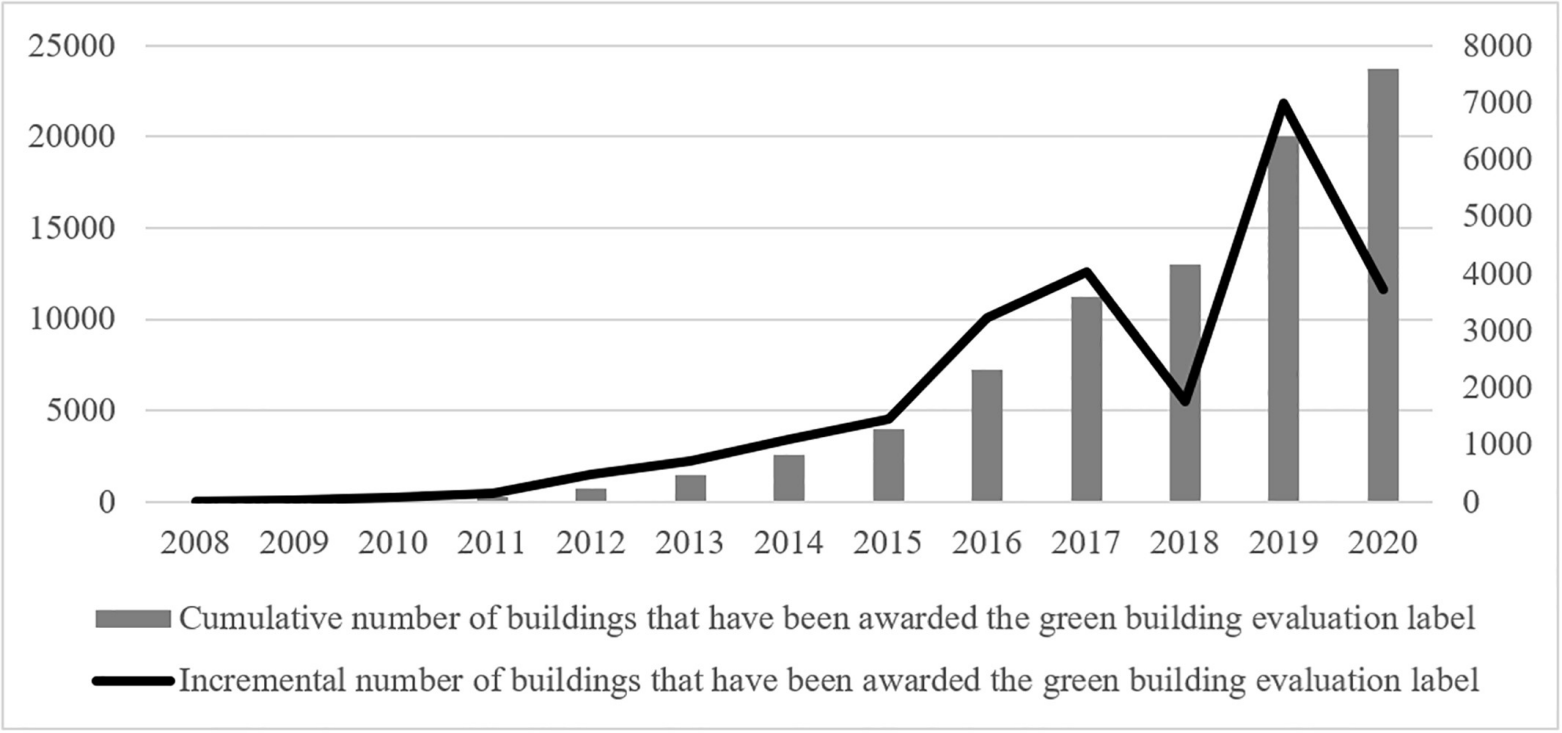
Growth trend of the number of green buildings in China.

**Fig 2:**
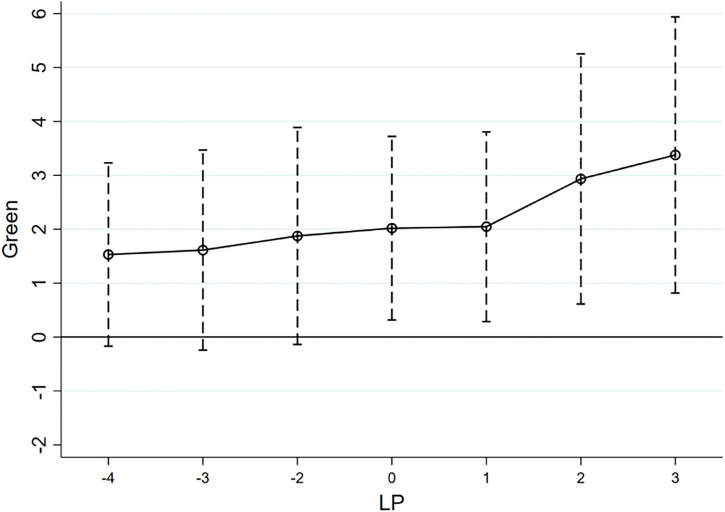
Parallel trend test results.

According to MOHURD data, green buildings in China reached a cumulative total of 8.5 billion square meters by 2021; the newly built green building area in the same year was 2 billion square meters, accounting for 84% of the newly built buildings. MOHURD had been carrying out an evaluation of the labeling of green building projects since 2008, but this work stopped in 2015. We recorded the number of buildings that were given the label at the national level from 2008 to 2020 and at the prefecture-city level from 2008 to 2015 and plotted these in Figs 1 and 2, respectively.

The bar chart and line graph in Fig 1 show the incremental and cumulative numbers of buildings, respectively, that have received green building evaluation labels in China from 2008 to 2020. As can be seen in Fig 1, the number of green buildings in China was low before 2012 and grew quickly after 2012. Although the incremental increase in green buildings dropped in 2018 and 2020, the total number of green buildings exhibits a faster growth trend from as early as 2008. This indicates the great importance that the Chinese government attaches to the promotion of green building.

### 3.2 Low-carbon city pilot

China’s National Development and Reform Commission (NDRC) issued the Circular on Pilot Work on Low-Carbon Provinces and Cities in 2010. This commission organized the pilot work for the LCPP, leading to the establishment of the first batch of pilot cities for low-carbon transition. The NDRC subsequently issued the Circular on the Second Batch of Pilot Work on Low-Carbon Provinces and Cities in 2012, establishing the second batch of low-carbon pilot cities. The cities included in the two low-carbon city pilots in 2010 and 2012 are showed in Table 1.

**Table 1:** Provinces and Cities for LCPP.

Year of policy implementation	Name of pilot city
2010	Five provinces (Guangdong, Liaoning, Hubei, Shaanxi and Yunnan) and eight cities (Tianjin, Chongqing, Shenzhen, Xiamen, Hangzhou, Nanchang, Guiyang and Baoding)
2012	Shijiazhuang, Qinhuangdao, Jincheng, Hulunbeier, Jilin, Daxinganling, Suzhou, Huaian, Zhenjiang, Ningbo, Wenzhou, Chizhou, Nanping, Jingdezhen, Ganzhou, Qingdao, Jiyuan, Wuhan, Guangzhou, Guilin, Guangyuan, Zunyi, Kunming, Yan’an, Jinchang and Urumqi.

Compared with other types of environmental policies, the LCPP is comprehensive and uses command-and-control, market-based, and voluntary policy instruments in its implementation. This policy aims to decarbonize city development by popularizing the concept of a low-carbon economy, adjusting the industrial structure, and improving energy efficiency. In the implementation of the LCPP, the central government only gives guidelines to the pilot areas, while the local governments carry out the pilots according to the guidelines and in accordance with the local realities.

The priorities of the pilot areas include low-carbon industrial development, optimization of energy structure, energy conservation and efficiency, increasing carbon sinks, and advocating low-carbon lifestyles. Specifically, the LCPP requires each city to prepare a low-carbon development plan based on its own resource endowment, industrial and energy structure, and economic development and to clearly set out the goals, tasks, and specific measures for reducing greenhouse gas emissions. In addition, local governments have adopted command-type policy tools for reducing and eliminating outdated production capacity and green building energy efficiency. These measures have been employed to constrain corporate carbon emissions, promote the reduction of corporate carbon emissions, and develop low-carbon industries. Local governments have implemented a target-responsibility system for controlling greenhouse gas emissions and have explored effective government guidance and economic incentives policies. Local governments have also begun to use market-oriented tools such as prices, financial subsidies, broadened financing channels, and tax incentives to ensure the smooth implementation of the policy, thereby promoting the reduction of regional carbon emissions.

## 4 Theoretical analysis and hypothesis

Currently, traditional buildings still dominate the construction market. The large upfront investment required and the slow conversion of benefits in green buildings mean that developing green buildings has a much higher cost than developing ordinary buildings, decreasing the willingness of developers to build green buildings in the market economy [59]. In addition, by investing in green buildings, developers both increase their own profits and enhance the public interest, increasing the energy-saving and environmental benefits enjoyed by residents.

As a result, the benefits of green buildings cannot be fully capitalized in the final transaction price, and their additional general and operating costs are usually higher than those of traditional buildings, with a mismatch between costs and benefits. This will lead to market failures and disincentives for real estate companies to supply green buildings [60]. In addition, regional energy efficiency and emissions-reduction programs are not taken into consideration by developers and consumers. The rational public is more concerned with price than the building’s green features when purchasing a house. Green buildings are more expensive than ordinary buildings, leading to lower purchase incentives for the public [61].

Due to the failure of the green building market, the government needs to introduce appropriate measures to intervene and promote the optimal supply of green buildings. Granderson et al. used the 1990 Clean Air Act in the United States, finding that governmental environmental regulatory policies promote green development [62]. Rubashkina et al. used European data to identify the positive effects of environmental policies on pollution reduction [63]. Many studies also identify the effectiveness of China’s LCPP in reducing carbon emissions. Yu et al. analyzed the impacts of LCPP from the perspective of carbon emissions and found that this policy is conducive to reducing carbon emissions [64]. In the case of the green building sector, studies find that public policies, including mandates and incentives, play a key role in promoting the GBD [45,46,65]. Zou et al. also found that the government’s subsidy policies in different regions of China can influence the spatial distribution of green buildings [48]. Therefore, we argue that, as an important policy of government environmental regulation, China’s LCPP can promote the GBD. Based on the above analysis, the first hypothesis of this study is as follows:

**H1** LCPP promotes the GBD.

Firm innovation activities are characterized by long cycles, large investments, and high risks. Technological innovation is fundamental to realizing green development. Porter’s hypothesis states that appropriate environmental regulation policies can stimulate technological innovation. The LCPP will force the government to adopt stricter environmental regulations to promote energy saving and carbon reduction and set stricter environmental standards, thereby increasing firms’ production costs. Firms seeking to maximize their profits will increase their R&D investment in innovation to improve productivity and competitiveness [66,67].

Pilot cities also experience transformation and diffusion of innovation achievements, incentivize low-carbon-technology research and development (R&D), introduce energy-saving and environmental protection technologies, and increase financial subsidies for clean and environmentally friendly production projects. This incentivizes firms to accelerate the innovation and development of green production technologies [68,69].

In addition, pilot cities have introduced market-based environmental regulatory tools, such as green financial policies and carbon emission subsidies, to reduce the cost of innovation and induce green technology innovation [70]. Innovation in green building technology is indispensable if the construction industry is to realize clean and green development. The level of investment required and the considerable uncertainty of R&D activities mean there is a certain degree of market failure in the process of technological innovation. Therefore, an increase in the level of regional green innovation will promote green investment by firms and promote the development of regional green buildings. Based on the above analysis, the second hypothesis of this study is as follows:

**H2** The low-carbon pilot city policy improves green innovation capacity, thus promoting the GBD.

Green total factor productivity (GTFP) is a comprehensive indicator that includes growth efficiency and resource and environmental factors. It incorporates non-desired outputs, such as resource consumption and environmental pollution, into the measurement framework. GTFP is a comprehensive evaluation of the degree of coordination between the economy and the environment and is an important indicator of green development [71,72]. There are many studies that uses green total factor productivity to measure high-quality development.

As an environmental policy, the LCPP can prompt firms to take resources and the environment as important factors for green production and force polluting firms to actively change their production methods, adjust their production structures, increase green production, and thereby improve their GTFP [73]. In addition, the core requirement of the LCPP is to improve production efficiency and reduce pollution emissions. The agglomeration effect resulting from the clustering of economic activities can be facilitated by increased productivity [71,72]. Adequate economic agglomeration can reduce the marginal cost of investment, thus encouraging green investment and increasing the firm’s GTFP [24,74].

At the regional level, Porter’s hypothesis suggests that environmental policies can force polluting firms to pursue technological innovation, improve green production efficiency, and shift from high energy-consuming and high-polluting production to green production methods. Environmental regulation can also eliminate polluting firms and expand the market share of green firms, adjusting the regional industrial structure and promoting the green development of the region [75].

As an important environmental policy, at the regional level, the LCPP can increase low-pollution and low-energy-consuming green investment activities, reduce regional pollution emissions and energy consumption, and thus enhance GTFP at the regional level [76]. In addition, the aggregation of economic activities stimulated by the low-carbon pilot policy is conducive to the formation of industrial synergies, which improves productivity and stimulates the competitive dynamic among firms, thus increasing the regional GTFP [77]. Further, the improvement of GTFP can significantly improve the efficiency of green investment by firms, reduce the cost, and incentivize firms to make more green investments. An increase in green total factor productivity implies an accelerated degree of green development in regions and enterprises, which can incentivize enterprises to invest more in green buildings and provide more green building supply. As an important green investment project for firms in the construction and real estate industry, the improvement of GTFP can promote GBD. Therefore, we propose the following third hypothesis:

**H3** LCPP improves GTFP, thus promoting the GBD.

The implementation of the LCPP has led local governments to introduce green financial policies with the autonomy granted by the central government. The government has, with the cooperation of financial institutions, broadened financing channels for firms in the construction and real estate industry through tax incentives, subsidies, and special funds, essentially lowering the credit threshold for green investment by firms [76,78].

Green finance actively guides the flow of credit funds from highly polluting and energy-consuming industries to clean and environmentally friendly green industries [74]. In addition, the policy incentivizes firms to engage in green production and investment by lowering interest rates and granting more loan funds. Further, it penalizes firms engaging in high-pollution and high-energy-consumption industries by restricting loans to polluting firms and increasing interest rates, thus making it more difficult for them to raise funds [23,79].

The LCPP reduces the financing cost and difficulty of firms engaged in green production and green investment so that firms investing in green projects can obtain credit preferences from the government. This policy guides capital allocation, leading firms to invest more capital in the construction of green buildings. The LCPP can reduce the financing constraints of firms in the construction and real estate industries, prompting them to invest funds in environmentally friendly green buildings, which promotes GBD. The fourth hypothesis of this study is as follows:

**H4** The LCPP reduces the financing constraints of firms, thus promoting the GBD.

## 5. Empirical design

### 5.1 Model

Since China’s LCPP was carried out in stages, we employ the staggered DID model to assess its impact on GBD. Studies have been conducted to examine the effects of public policies using two main approaches: the fixed effects model and the difference-in-differences (DID) model. Compared with the fixed effects model, the DID model can effectively identify causal relationships and is therefore widely used in studies that evaluate policy [80]. The extant studies have mostly designed the DID model to identify the effects of green credit policy or green tax policy on macroeconomic and micro subjects. Since the LCPP is gradually rolled out and implemented in phases, this paper employs the staggered DID model to identify the effect of LCPP on GBD. The staggered DID model is expressed as follows:

$$Green_{i,t}=\beta_{0}+\beta_{1}LC_{it}+\gamma X_{i,t}+\theta_{i}+\delta_{t}+\varepsilon_{i,t}$$ (1)

where *Green*_*i*,*t*_ represents green building development in the prefecture-level city i in year *t* and *LC*_*it*_ is a dummy variable that denotes whether city i is a pilot city in year t. *X*_*i*,*t*_ is a series of prefecture-level control variables that may affect the GBD. We also control prefecture-level city fixed effect (*θ*_*i*_) and year fixed effect (*δ*_*t*_). Standard errors are clustered at the prefecture level. We are interested in the coefficient *β*_1_, which measures the impact of LCPP on GBD.

### 5.2 Data and variables

#### 5.2.1 Data

**Green Building Data.** This data comes from the Green Building Evaluation and Labeling Program announcements published in the “Green Building Evaluation and Labeling Network,” established by the MOHURD in cooperation with the Ministry of Science and Technology, as well as the public notices of all the green building evaluation and labeling programs published by the MOHURD. In this study, green buildings refer to buildings given the green building label. MOHURD has been carrying out the evaluation of green building labeling projects since 2008 and stopped the work in 2015. We collected data on new green buildings in prefecture-level cities in 2008–2015. In addition, we exclude four municipalities directly under the central government and some prefecture-level cities with missing data; our final sample comprises a total of 2,322 green building projects covering 206 prefecture-level cities.

**Low-carbon city pilot data.** NDRC started the first batch of LCPP in 2010 and carried out the second batch of the pilot in 2012.

**Prefecture-level data.** A series of prefecture-level control variables are introduced in the baseline regression, and four prefecture-level mechanism variables are also introduced in the mechanism analysis. The above data come from the China Urban Statistical Yearbook, China Environmental Statistical Yearbook, China Labor Statistical Yearbook, and the statistical yearbooks of each province.

**Firm-level data.** In the mechanism analysis, we match prefecture-level data with firm-level data to analyze the impact of LCPP on GBD. Firm-level data obtained from China Stock Market & Accounting Research (CSMAR) Database. This database is a comprehensive research-oriented database focusing on China Finance and Economy, and is widely used in the study of Chinese companies. We use the merge command of the Stata software to match firm-level data with prefecture-level data by the administrative code of the firm’s location.

We process the samples as follows: (1) we exclude prefecture-level cities with missing samples; (2) we exclude four municipalities directly under the central government; and (3) We winsorized all the continuous variables at the 1% and 99% levels to exclude the outlier effect.

#### 5.2.2 Variables. Dependent variable

The dependent variable is green building development (*Green*). The number of green building projects represents the level of regional GBD. Green buildings in China need to be certified by the government. A project that obtains the green building label and is publicized on the official website of the MOHURD is defined as a green building. Therefore, we measure GBD by the number of green building labels assigned.

**Core independent variable**. The core explanatory variable is a dummy variable for low-carbon city pilot policy (*LP*). We use the time-series difference in the approval of the LCPP in each prefecture-level city to measure low-carbon city construction. We employ the LCPP as a quasi-natural experiment. The first and second batches of LCPP in China were approved in October 2010 and December 2012, respectively, both near the end of the year. Considering the lag in policy implementation, we define the policy implementation times as 2011 and 2013. Specifically, the dummy variable takes the value of 1 in the year when the prefecture-level city is approved as a low-carbon city pilot and in subsequent years, and 0 otherwise.

**Mechanism variables**. Green Innovation (*Innovation*). We measure this variable using the natural logarithm of the number of patents for green inventions.

Green total factor productivity (*GTFP*). We calculate the GTFP at the firm and regional levels separately. For the GTFP at the firm level (*GTFP_firm*), we incorporate firm environmental pollution into the evaluation and adopt the non-radial SBM-ML index to measure the GTFP of firms. The measurement of input and output indexes of firm GTFP includes factor inputs and desired and non-desired outputs. Factor inputs include inputs of labor, capital, and energy. Labor input is measured by the number of employees in the firm; capital input by the net fixed assets of the firm, and energy input is measured by the industrial electricity consumption of the city where the firm is located, converted by the proportion of the firm’s employees in the employment of urban workers in the city. Desired output is measured by business revenue. The non-expected output is converted to emissions of industrial sulfur dioxide, industrial wastewater, and industrial fumes and dust by the share of firms’ employees in the employment of the urban population of the city in which they are located.

In addition to the GTFP at the firm level, we follow Pastor et al., Tone, and Oh and Heshmati and measure the GTFP of prefectural-level cities (*GTFP_city*) using the super-efficiency SBM model of non-expected output and the Malmquist productivity index under the framework of the data envelopment analysis of global reference [81–83]. Labor input is measured by the employed population of each city, capital input by the capital stock calculated by the perpetual inventory method, energy input by electricity consumption, desired output by real GDP deflated by the GDP deflator (with 2008 as the base period), and non-desired output by the emissions of greenhouse gases and environmental pollutants.

Financing constraints (*WW*). We use the WW index to measure firms’ financing constraints. The WW index is constructed by parameter estimation of the investment Euler equation using the system generalized moments GMM (Whited and Wu, 2006), which is modeled as follows:

$$WW=-0.091CF_{it}-0.062DIV_{it}+0.021Lev_{it}-0.044Size_{it}+0.102ISG_{it}-0.035SG_{it}$$ (2)

where *CF* is measured by the ratio of net cash flow from operating activities to total assets; *DIV* is a dummy variable for whether or not dividends are paid, which is 1 when the firm pays out cash dividends, and 0 otherwise; *Lev* is the gearing ratio, which is the ratio of total liabilities to total assets; *Size* is the natural logarithm of the firm’s total assets, *ISG* is the growth rate of the industry’s operating income, and *SG* is the growth rate of the firm’s operating income. The larger the WW index, the higher the level of financing constraints faced by the firm.

**Control variables**. Following existing studies, the control variables include the level of regional economic development (*Per_GDP*), the proportion of the tertiary industry (*Industry*), the logarithm of the number of construction and real estate employees (*Employ*), the logarithm of population density (*Density*), the logarithm of real estate development investment completion (*Investment*), and logarithm of local financial general budget revenue (*Revenue*). The definition of each variable is shown in Table 2[13,26,48,84,85].

**Table 2:** Variable definitions.

Variables	Definitions
*Depedent variables*	
*Green*	Natural logarithm of the number of green building labels obtained.
*Key independent variable*	
*LP*	Dummy variables. This variable takes the value of 1 in the year a prefecture-level city is approved as a low-carbon city pilot and in subsequent years, and 0 otherwise.
*Mechanism analysis variables*	
*Innovation*	Logarithm of the number of green patents filed by the firm
*GTPF_firm*	Measured using the non-radial SBM-ML index
*GTPF_city*	Measured using the super-efficient SBM model with non-expected output and the Malmquist productivity index
*WW*	The WW index is constructed by parameter estimation of the investment Euler equation using the system generalized moments GMM [86], and the calculation method is shown in Eq (2).
*Control variables*	
*Per_GDP*	Natural logarithm of GDP per capita.
*Industry*	Value added of the tertiary sector as a share of GDP
*Employ*	Logarithm of the number of people employed in construction and real estate
*Density*	Logarithm of population density
*Investment*	Logarithm of real estate development investment completion
*Revenue*	Logarithm of local financial income from the general budget

### 5.3 Descriptive statistics

The descriptive statistics of the variables are shown in Table 3. Table 3 shows that the mean value of Green is 0.734, but the standard deviation is 2.041, which indicates that the number of green buildings in China is not large, and the number of green buildings varies greatly between regions, and the development is relatively unbalanced.

**Table 3:** Descriptive statistics of main variables.

Variables	Observations	Mean	St. Dev	Min.	Max.
*Green*	2250	0.734	2.041	0	17
*LP*	2250	0.175	0.380	0	1
*Innovation*	1868	2.195	1.562	0	6.444
*GTPF_firm*	859	0.871	0.065	0.751	0.978
*GTPF_city*	2234	0.993	0.031	0.811	1.100
*WW*	859	-0.832	0.534	-1.191	-0.752
*Per_GDP*	2250	16.181	0.869	14.306	18.378
*Industry*	2250	0.365	0.086	0.175	0.653
*Employ*	2250	1.578	0.874	0.166	4.020
*Density*	2250	5.707	0.887	2.890	7.148
*Investment*	2250	13.720	1.189	10.908	16.640
*Revenue*	2250	13.478	1.023	11.191	16.138

Meanwhile, the mean value of *LP* is 0.175, which indicates that the proportion of low-carbon city pilots is not high among all cities. In addition, the mean values of the four mechanism variables of Innovation, *GTPF_firm*, *GTPF_city* and *WW* are 2.195, 0.871, 0.993 and -0.832 respectively, which are consistent with the mean values of the relevant variables in the existing studies, and once again proves the accuracy of the data in this study.

## 6. Results

### 6.1 Baseline results

Table 4 reports the results of the baseline regressions. Column (1) does not introduce any control variables and controls only city fixed effects. Column (2) introduces control variables based on Column (1), while Column (3) further introduces year fixed effects. The coefficients of *LP* in Columns (1)-(3) are 1.118, 1.008, and 0.899, respectively, and all of them are significant at the 1% statistical level, indicating that the LCPP significantly promotes the GBD, which verifies Hypothesis 1. In addition, the coefficient of *Employ* in Column (3) is also significantly positive at 1% statistical level, indicating that the increase in the number of regional construction and real estate employees also significantly promotes the GBD. This suggests that the development of the real estate sector can significantly contribute to the development of green buildings.

**Table 4:** Baseline results.

Variables	(1)	(2)	(3)
	Dependent variable: *Green*		
*LP*	1.881*** (0.320)	1.008*** (0.334)	0.899*** (0.312)
*Per_GDP*		0.684 (0.512)	-0.954 (1.342)
*Industry*		8.265*** (1.579)	2.955 (2.595)
*Employ*		0.749*** (0.234)	0.687*** (0.244)
*Density*		0.237 (0.342)	0.303 (0.394)
*Investment*		0.084 (0.133)	0.065 (0.114)
*Revenue*		-0.092 (0.280)	-0.258 (0.299)
Constant		-15.958*** (5.148)	14.745 (22.035)
City FE	Yes	Yes	Yes
Year FE	No	No	Yes
Observations	2305	2250	2250
R^2^	0.4641	0.5213	0.5281

Note: The standard deviation is reported in the parentheses.

*, **, and *** indicate significance at the 10%, 5%, and 1% levels, respectively.

The baseline results show that LCPP promotes GBD, which supports the extant studies on the impact of green policies on green buildings. Extant studies have examined the impact of public policies supporting green development, such as green finance, green taxes, and green subsidies, on green buildings, and have found that these policies play a positive role in green development [53,55]. Our findings demonstrate the contribution of the green policy of LCPP to GBD, and enrich the studies related to the effects of green public policies.

### 6.2 Robustness checks

#### 6.2.1 Parallel-trends test

The use of the DID model to evaluate the policy effect requires that the level of green building development in the treatment and control groups follow the same trend before the policy shock. We thus test whether the level of green building development satisfies the parallel trends assumption. Following Beck et al, we adopt the event study to analyze the trends between the treatment and control groups and establish the following model [87]:

$$Green_{it}=\gamma +\sum_{k=-M}^{N}\gamma_{j}{\text{LC}}_{i,t-k}+\tau Control_{it}+\gamma_{i}+\delta_{t}+\varepsilon_{it}$$ (3)

**Fig 3:**
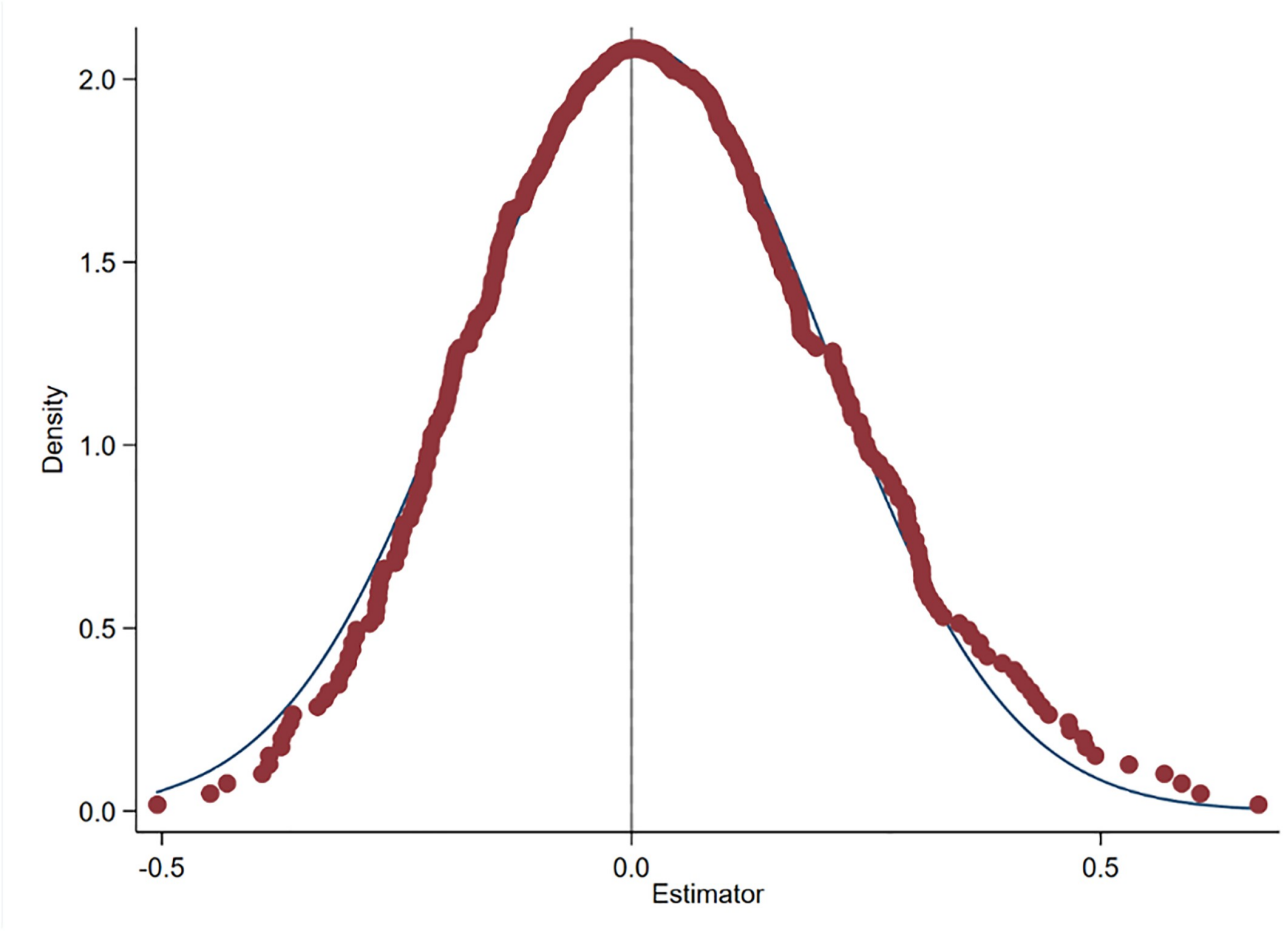
Placebo test results.

Based on the results of the event study, we plotted parallel trends, as shown in Fig 2. As can be seen in Fig 2, the 95% confidence intervals of the coefficient estimates before the LCPP all contain zeros, indicating that none of these coefficients are significant, which suggests that there is no significant difference in the GBD in the treatment group and the control group before the implementation of the LCPP. In addition, after the implementation of the LCPP, none of the 95% confidence intervals of the coefficient estimates include 0, indicating that the estimated coefficients are significant, which further suggests that the GBD is promoted in the year of the LCPP as well as in subsequent years. Further, the value of the estimated coefficients continues to increase after the implementation of the LCPP, indicating that the policy has played a continuous role in promoting the GBD. The results depicted in Fig 3 support the parallel-trend assumption of the DID method, indicating the robustness of the baseline regression results.

#### 6.2.2 Placebo test

We introduce a series of prefecture-level control variables to the baseline regression to mitigate the impact of omitted variables on GBD. There may still be unobserved regional characteristics that have an impact on GBD, thus leading to biased results in the baseline regression. We exclude the impact of the LCPP on GBD from the interference of omitted variables with reference to Liu and Lu and conduct a placebo test by randomly selecting the pilot year of the LCPP and randomly generating the treatment group [88].

Using a randomly selected sample, we repeat the baseline regression 500 times. If the mean value of the regression coefficients of the new dummy variables is close to 0, this indicates that our results in the baseline regression are plausible. Fig 3 illustrates the results of the placebo test. The curves in Fig 3 are the kernel density distributions of the estimated coefficients of the dummy variables. The results of the 500 random-sample regressions show that the mean of the coefficients of the dummy estimates in the graphs is close to 0 and that the estimated coefficients of the baseline regression are completely independent of the distribution of this coefficient. This suggests that the impact of the LCPP on GBD is not disturbed by omitted variables.

#### 6.2.3 Excluding other policy shocks

If other policy shocks related to GBD occur within the sample interval of this study, they may also affect the accuracy of the baseline results. Within the sample interval of this study, the carbon emissions trading pilot policy and the innovative city pilot policy may affect the GBD. We exclude the impact of these policy shocks on the identification results by introducing a dummy variable to capture whether a city is affected by the carbon emissions trading pilot policy (*CE*) and the innovative city pilot policy (*IC*) in the baseline model.

Specifically, *CE* is assigned a value of 1 in the current year and in subsequent years when a region is affected by the carbon emissions trading pilot policy, and 0 otherwise. Similarly, *IC* is assigned a value of 1 for the current year and subsequent years in which a region is affected by the innovative policy, and 0 otherwise. The regression results in Columns (1) and (2) in Table 5 show that after introducing the dummy variables, the coefficients of *LP* for the two policies are 0.809 and 0.911, respectively; both are significant at the 1% level, which proves the robustness of the baseline results.

**Table 5:** Robustness check results.

Variables	(1)	(2)
	Dependent variable: *Green*	
*LP*	0.809	0.911
Controls	Yes	Yes
City FE	Yes	Yes
Year FE	Yes	Yes
Observations	2250	2250
R^2^	0.5294	0.5291

Note: The standard deviation is reported in the parentheses. *, **, and *** indicate significance at the 10%, 5%, and 1% levels, respectively.

#### 6.2.4 Heterogeneous treatment effects

The treatment groups in the staggered DID model are not exposed to policy shocks at the same time. Callaway and Sant’Anna and Baker et al. found that the estimates of a staggered DID model are weighted averages of the policy effects of the cohorts across periods, implying homogeneous treatment. Staggered DID estimates are essentially weighted averages of the effects of several different treatments, and there may be cases where the weights are negative [89,90].

In the case of negative weights, the average treatment effect obtained from the weighted average of the different treatment effects may be opposite to the true average treatment effect [90]. In addition, in practice, there is heterogeneity in the responses of different cities or firms to the same policy. The estimation of the baseline regression may be biased due to heterogeneous treatment effects when applying staggered DID identification. The true parameters estimated by the staggered DID can be treated as the expected value of the weighted sum of treatment effects for all treated units.

Following De Chaisemartin and D’Haultfoeuille, we compare the evolution of the outcomes between consecutive periods across pairs of groups [91]. However, the “control group” in some of those comparisons may be treated at both periods. Then, its treatment effect in the second period is differenced out by the DID, hence the negative weights. The staggered DID estimates may not be robust when the negative weights are large. The closer the heterogeneity treatment robustness index is to 1, the more robust the results are, and the closer to 0, the less robust. A test of the weights of the estimators shows the standard deviation of the metric estimates under the heterogeneous treatment effect is close to 0, indicating that there is a heterogeneous treatment effect in the estimates that may lead to a biased baseline result.

We use fuzzy DID to estimate the local average treatment effect for the change in status of a prefecture-level city to a low-carbon city pilot. The indicator variables are the change in the LCPP in two adjacent periods (*G_T*) and its first-order lag (*G_T-1*), respectively; the time variable is the year, and the treatment variable is the LCPP (*TT*). The empirical results show that the fuzzy DID estimate of the Wald-TC estimation is 0.787 and significantly negative at the 5% level. This shows that after considering the heterogeneous treatment effect, the local treatment effect for individuals with a change in treatment status is still significant, which confirms the robustness of the baseline results.

### 6.3 Mechanism analysis

We further introduce the interaction term between mechanism variables and core independent variables based on Model (1) to investigate the mechanism by which the LCPP affects the GBD and construct the following model:

$$Green_{i,t}=\beta_{0}+\beta_{1}LP_{it}\times Mechanism_{it}+\beta_{2}LP_{it}\times \beta_{3}Mechanism_{it}+\delta_{i}+\delta_{t}+\varepsilon_{i,t}$$ (4)

where *LP*_*it*_ × *Mechanism*_*it*_ is the interaction term between the mechanism variables and the core independent variables. The definitions of the other variables are consistent with model (1). The coefficient we are interested in is *β*_1_.

Based on our theoretical analysis, we select four mechanism variables: green technological innovation, GTFP at the regional and firm levels, and firm financing constraints. The definition of each variable is set out in Table 1. Since GBD is relevant to construction and real estate firms, we select listed companies in these industries when testing the mechanism at the firm level and matching the firm- and prefecture-level data in the baseline regression.

We introduce the four mechanism variables into Model (5) and regress each separately. The regression results are shown in Table 6. Column (1) shows the regression results of the green innovation mechanism; Column (2) and Column (3) show the regression results of the GTFP mechanism at the firm level and the regional level, respectively; and Column (4) shows the regression results of the firm financing constraint mechanism. The regression results show that in Column (1), the coefficient of *LP×Innovation* is 1.248 and is significant at the 1% level, indicating that green innovation significantly contributes to the impact of the LCPP on the GBD. The coefficients of *LP×GTPF_firm* and *LP×GTPF_city* in Columns (2)–(3) are 12.263 and 35.191 and are significant at the 10% and 5% levels, respectively. This indicates that the improvement of GTFP at the firm and regional level significantly enhances the promotion effect of the LCPP on GBD; the coefficient of *LP×WW* in Column (4) is −9.880 and is significant at the 5% level. Since the larger WW index indicates that firms face greater financing constraints, the result suggests that LCPP significantly reduces the financing constraints of firms in the construction and real estate industries, thus promoting the GBD. The above results show that LCPP significantly enhances the regional capacity for green innovation, improves the GTFP of firms and regions, and reduces firm financing constraints, thus promoting the GBD; Hypotheses 2–4 are verified.

**Table 6:** Mechanism analysis results.

Variables	(1)	(2)	(3)	(4)
	Dependent variable: *Green*			
*LP×Innovation*	1.248*** (0.190)			
*LP×GTPF_firm*			35.191* (19.619)	
*LP×GTPF_city*		12.263** (6.193)		
*LP×WW*				-8.800** (4.031)
*LP*	-2.504*** (0.419)	0.764*** (0.289)	-28.722 (17.452)	-4.106 (4.806)
*Innovation*	-0.092 (0.084)			
*GTPF_firm*			0.463 (7.351)	
*GTPF_city*		2.496** (1.198)		
*KZ*				0.787 (0.992)
*Controls*	Yes	Yes	Yes	Yes
Year FE	Yes	Yes	Yes	Yes
Industry FE	Yes	Yes	Yes	Yes
Observations	1860	2215	736	792
R^2^	0.6082	0.5165	0.8129	0.8407

Note: The standard deviation is reported in the parentheses.

*, **, and *** indicate significance at the 10%, 5%, and 1% levels, respectively.

The results of the mechanism test are consistent with the theoretical analysis and verify hypotheses 2–4. Further, we provide economic interpretations of these results. First, the LCPP promotes green innovation of firms, thus promoting GBD. The innovation of green building technology is indispensable for the construction industry to realize clean and green development [43]. The market failure in technological innovation exists due to the high investment and uncertainty of R&D activities. LCCP not only enhances the protection of patents and property rights, reduces the phenomenon of “free-riding” among firms, and increases the R&D investment of enterprises, but also subsidizes the green innovation activities of firms, and incentivizes firms to carry out green innovation.

Secondly, LCPP promotes GTFP at the regional and firm levels, which promotes GBD. GTFP is an important indicator for evaluating high-quality economic development. On the one hand, LCPP can promote high-quality economic development, of which the key connotation is green development. GTFP at the regional level, as an important indicator for measuring green development in the construction industry, can further promote GBD. On the other hand, GTFP at the firm level considers the negative externalities of economic growth. It incorporates environmental pollution as a non-desired output into the accounting framework and emphasizes the maximization of economic benefits and minimization of environmental pollution with minimum resource input. An increase in GTFP can significantly improve the green investment efficiency and reduce costs of firms, and incentivize them to make more green investments, thus promoting GBD.

Finally, LCPP reduces the financing constraints of firms, thus promoting GBD. After the implementation of the LCPP, local governments introduced green financial policies with the autonomy granted by their superiors. With the cooperation of financial institutions, firms take advantage of tax incentives, government subsidies, and special funds to broaden their financing channels. As a result, LCPP reduces the financing cost and difficulty for firms engaged in green production and investment, guides capital allocation, and incentivizes them to invest more capital in green buildings.

## 7. Further analysis

We further examine the heterogeneity effect of LCPP affecting GBD. In this study, we examine the heterogeneity of cities in three aspects: location, resource endowment, and city size. The results of the heterogeneity analysis are shown in Table 7.

**Table 7:** Heterogeneity analysis results.

Variables	(1)	(2)	(3)	(4)	(5)	(6)	(7)
	Eastern	Central	Western	Resource cities	Non-resource	Large	Small
	Dependent variable: *Green*						
*LP*	0.577** (0.277)	0.963*** (0.323)	2.793*** (0.764)	0.837*** (0.317)	0.909*** (0.314)	2.114*** (0.574)	0.035 (0.135)
*Controls*	Yes	Yes	Yes	Yes	Yes	Yes	Yes
Year FE	Yes	Yes	Yes	Yes	Yes	Yes	Yes
Industry FE	Yes	Yes	Yes	Yes	Yes	Yes	Yes
Observations	704	492	879	1348	902	1125	1125
R^2^	0.4499	0.5141	0.6077	0.5059	0.5098	0.5593	0.3085

Note: The standard deviation is reported in the parentheses.

*, **, and *** indicate significance at the 10%, 5%, and 1% levels, respectively.

### 7.1 Heterogeneity of city location

There are large differences in the level of economic development, intensity of policy implementation, and historical and cultural characteristics of cities in different areas of China, which may lead to differences in the effect of the LCPP on GBD. Therefore, we examine this impact in different areas. We divide the full sample into eastern, central, and western regions according to the standards of the National Bureau of Statistics of China and then conduct group regression in the sub-sample. Columns (1)-(3) of Table 7 show the regression results of city location heterogeneity. In Columns (1)-(3), the coefficients of LP are 0.577, 0.963, and 2.793, and are significant at 5%, 1%, and 1% statistical levels, respectively. The result indicates that the LCPP promotes the GBD in the eastern, central, and western regions of China.

In addition, the coefficient value of *LP* is largest in the western region and smallest in the eastern region, indicating that the LCPP plays the largest role in promoting GBD in the former and the smallest role in the latter. When the LCPP that incentivizes GBD is implemented, it has a greater marginal effect on the growth of green buildings in the western region and, thus, a greater positive impact on GBD.

### 7.2 Heterogeneity of city resource endowment

We follow the division criteria in China’s Sustainable Development Plan for Resource-based Cities (2013–2020) and divide the whole sample into resource-based and non-resource-based cities. We regress these based on Model (1) to analyze the differences in the impacts of the LCPP on the GBD in different resource-endowed areas. Columns (4)-(5) of Table 7 show the regression results of the heterogeneity of city resource endowment. In Columns (4)-(5), the coefficients of *LP* are 0.837 and 0.909, respectively, and both are significant at the 1% statistical level, indicating that LCPP can promote the GBD in both resource-endowed and non-resource-endowed cities. Meanwhile, the coefficient of *LP* in Column (4) is smaller than that in Column (5), indicating that LCPP has a greater impact on the GBD in non-resource cities.

The LCPP requires regions to adjust their energy structure, reduce high energy-consuming and high-polluting firms, increase the use of clean energy, and carry out industrial restructuring. In resource-based cities, natural resources are an important source of economic development, and firms rely on their resource endowments for production. At this point, the LCPP has prompted firms in resource-based cities to change their production methods to a greater extent than firms in non-resource-based cities, resulting in higher costs and more time to improve their existing energy and product structures. Therefore, the LCPP has a lesser effect on the enhancement of firms in cities with higher resource dependence compared with firms in cities with lower resource dependence. As a result, the LCPP has a weaker role in promoting the GBD in resource-based regions than in non-resource-based regions.

### 7.3 Heterogeneity of city size

City size may have an impact on regional pollution emissions, human capital, and industrial agglomeration, impacting the promotion effect of the LCPP on GBD. Therefore, we divide the full sample into large- and small-scale cities according to regional population and perform group regressions. The regression results are shown in Columns (6)-(7) of Table 7. The results show that the coefficients of *LP* in Columns (6)-(7) are 2.144 and 0.035, respectively, and the former is significant at the 1% statistical level, while the latter is not, indicating that the LCPP can significantly promote the GBD in large-scale cities, but does not have a significant impact on the GBD in small-scale cities. The population agglomeration of large-scale cities can guarantee the effective implementation of the LCPP, which is more conducive to the LCPP and thus has a greater effect on the promotion of GBD.

## 8. Conclusions

This study is the first theoretical and empirical examination of the impact of the LCPP on GBD. The conclusions of this study are as follows: (1) The LCPP in China significantly promotes GBD, and this result remains robust after parallel trends, placebo, other-policy-shock, and heterogeneity-treatment-effect tests; (2) The LCPP significantly enhances regional green innovation capacity, improves regional and firm GTFP, and reduces the financing constraints of firms in the construction and real estate industries, which in turn promotes the GBD; and (3) The promotion effect of LCPP on GBD is greater in the central and western regions of China and in resource-oriented and large-scale cities.

Based on the above findings, we make the following policy recommendations. First, the LCPP should be further implemented, and the pilot program should be expanded. The LCPP significantly promotes the GDB and reduces carbon emissions. This is conducive to the reduction of pollution and carbon emissions in the region but also significantly reduces the emissions of neighboring cities because of the spatial spillover effect. The policy’s emission reduction effect should be fully realized, and the policy gradually extended to other non-pilot cities by comprehensively considering the development situation of each city. Second, regional green innovation capacity should be improved. The government should further improve the role of firms as market subjects in green technological innovation and low-carbon industrial transformation and adopt a variety of policies to incentivize firm’s green technological innovation capacity.

Third, GTFP should be enhanced. The government should also pay greater attention to environmental protection in economic development, maximize green economic growth, and incentivize firms to engage in more green production and investment, thereby improving GTFP at the firm and regional levels. Fourth, supporting credit policies should be introduced to reduce the financing constraints of firms in the construction and real estate industries. The credit constraints of these firms are an important factor affecting their investment activities. It is necessary to offer credit support for construction and real estate firms engaging in green investment activities to reduce their borrowing thresholds and costs. Fifth, the LCPP should be oriented toward the central, western, and resource-rich regions. Policymakers should tilt LCPP toward cities in central and western regions, as well as those with high resource dependence, and provide greater guidance and support.

This study helps to promote the GBD, reduce carbon emissions, and provide a reference for the realization of green and sustainable development in China. It also provides references for carbon reduction in other developing countries that must address their carbon emissions and environmental pollution. In addition, due to the market failure of green building, government intervention is needed to realize the optimal allocation of resources. Therefore, future research could focus on other government interventions, such as green credit policies, financial subsidies, and tax incentives, to analyze the impact of these policies on the GBD.
